# Looking ahead in working memory to guide sequential behaviour

**DOI:** 10.1016/j.cub.2021.04.063

**Published:** 2021-06-21

**Authors:** Freek van Ede, Jovana Deden, Anna C. Nobre

**Affiliations:** 1 Oxford Centre for Human Brain Activity, Wellcome Centre for Integrative Neuroimaging, Department of Psychiatry, University of Oxford, Oxford, UK; 2 Institute for Brain and Behavior Amsterdam, Department of Experimental and Applied Psychology, Vrije Universiteit Amsterdam, Amsterdam, The Netherlands; 3 Department of Experimental Psychology, University of Oxford, Oxford, UK

## Abstract

Working memory can maintain multiple sensory representations to serve unfolding sequential behaviour, such as while making tea or planning a route. How the human mind juggles internal representations as they become relevant to guide sequential behaviour remains poorly understood. Specifically, while there is good evidence that we can flexibly switch priorities among representations in working memory^1^, ^2^, ^3^, ^4^, it is unclear how and when dormant memory representations are brought into focus during sequential behaviour. Capitalising on a recently established and temporally precise gaze marker of internal selection^5^^,^^6^, we reveal that the focus in the mind moves to the next-relevant memory representation while behaviour associated with the presently relevant memory representation is still ongoing. Thus, like visual sampling of external objects in the world^7^, ^8^, ^9^, internal visual sampling also ‘looks ahead’ to the next object in memory during sequential behaviour.

## Teaser

van Ede *et al.* show that, when sequential behaviour is guided by internal visual representations, the focus in mind ‘looks ahead’ to the next object in memory while behaviour associated with the preceding object is still ongoing.

## Main text

**Figure 1:**
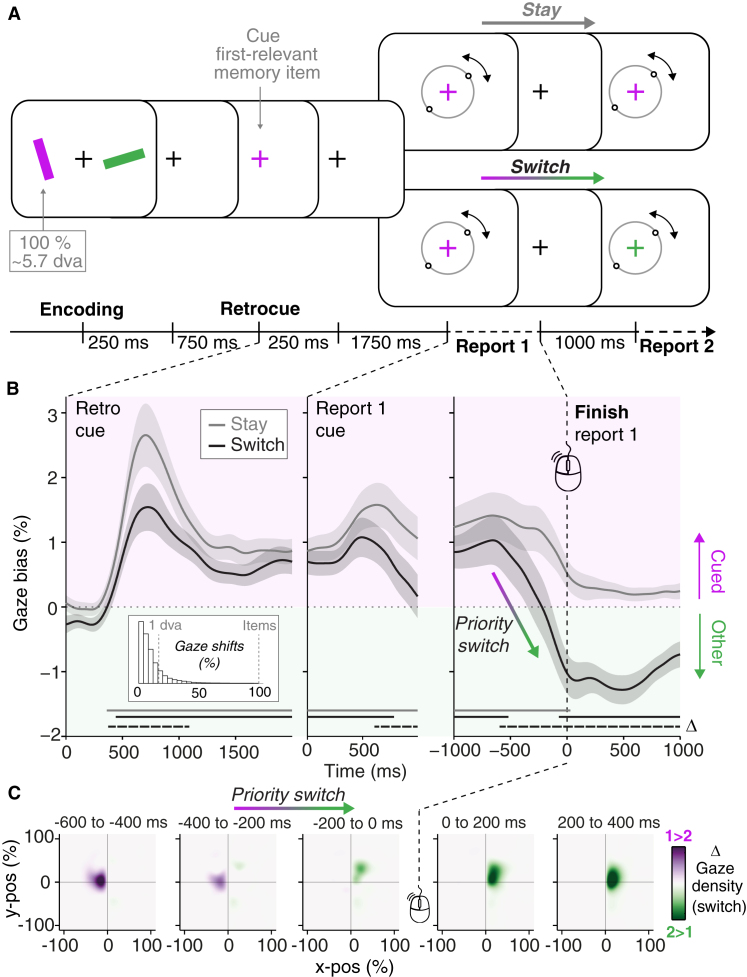
Focusing ahead in visual working memory during sequential behaviour. (A) Participants remembered the colour and tilt of two bars for sequential reproduction. A retrocue informed which bar would be probed first. In switch blocks, participants subsequently reported both bars in sequence, requiring an internal switch of priority. (B) Gaze bias toward the memorised locations of the two representations, aligned to three key moments in the trial. Gaze bias in percentage, with 100% denoting the centre of the items at encoding (5.7 degrees visual angle). Horizontal lines denote significant clusters (dashed lines denote switch vs. stay). Shading represents ± SEM. Inset shows the distribution of gaze-shift magnitudes for all detected gaze shifts (n = 48932) accumulated over the depicted intervals (‘1 dva’ refers to one degree visual angle). Gaze shifts were identified using a median-based threshold on gaze velocity time courses. (C) Heatmaps of gaze density in switch trials showing when gaze is more biased toward the location of the first-relevant or second-relevant memory item, aligned to report-1 completion.

Twenty-five human volunteers performed a visual working-memory task; memorising green and purple bars to later report their precise orientation from memory (Figure 1A). Reporting involved matching the orientation of a central response dial to that of the probed bar in memory. Dial-up was controlled by the computer mouse and terminated by a mouse click. In most trials a fixation-cross colour change (retrocue) during memory maintenance drew attention to the item that had to be reported first. In *stay blocks*, memory for the same item was probed twice in sequence (allowing participants to drop the other item from memory). In contrast, in the crucial *switch blocks*, the other item would always be probed next, requiring it to move into the focus of attention to guide the second report.

To track when the internal focus switched between the two memory items with high temporal precision, we capitalised on a gaze marker of internal selection that we recently established^5^^,^^6^. For a fuller characterisation of this marker, and its relation to related literature, see our recent article dedicated to this gaze bias^5^: in short, it consists of a directional bias in fixational eye movements known as microsaccades^10^ (see inset in Figure 1B for the distribution of gaze shifts in the current dataset) and can be visualised and quantified as a change in gaze position over time.

After the retrocue, gaze position in both block types became biased in the direction of the memorised location of the cued memory item (Figure 1B, left panel; cluster p_*stay*_ < 0.0001; cluster p_*switch*_ < 0.0001), comparable to our previous demonstrations of this gaze bias^5^^,^^6^. At the same time, we observed that this gaze bias to the first-relevant memory item was attenuated in switch compared to stay blocks (cluster *p* = 0.0023). This occurred despite the observation that retrocues benefitted performance on the first report similarly in both block types (Figure S1 in the Supplemental information). This relative attenuation of gaze bias after the retrocue in switch blocks likely reflected the continued relevance of the other memory representation for later use in switch trials.

The most interesting and unexpected finding occurred when aligning the gaze data to the end of the first memory-guided report (completed 1428 ± 50 ms and 1416 ± 47 ms after report-cue onset in stay and switch trials, respectively). Intuitively, the focus in working memory should shift to the next item only once action guidance by the preceding item is completed. Contrary to this intuition, our gaze marker of internal focusing revealed that the internal priority switch started well before report 1 was completed (Figure 1B, right panel). Gaze-bias direction in switch and stay blocks started to differ approximately 600 ms (first significant difference at –596 ms) *prior* to completion of report 1 (cluster p < 0.0001), with gaze bias reversing direction in switch trials, but not in stay trials.

Following the retrocue — which triggered the *initial* prioritisation in memory — gaze bias reached its maximal deflection 484 ms later. If the priority switch to the next-relevant memory item commenced after report 1 completion, one would expect to find the maximal deflection associated with the priority switch at similar latencies after report 1. Instead, the gaze bias reversal in switch trials reached its maximum deflection 120 ms *prior* to the end of report 1 (approximately 600 ms earlier). In fact, by the time participants clicked the mouse button to complete report 1, gaze was already significantly biased in the opposite direction; toward the memorised item that would be needed next (cluster p < 0.0001). This was also evident when visualising time-resolved heatmaps of gaze position in switch trials (Figure 1C).

The central looking-ahead pattern associated with internal priority switching in working memory could also be demonstrated using complementary analyses of gaze-shift rates and landing positions (Figure S2A,B), and occurred regardless of dial-up duration (Figure S2C) or whether report 1 could be anticipated (Figure S2D). Moreover, the gaze bias associated with this priority switch predicted performance of report 2 (Figure S2E).

When guiding sequential actions by visual objects in the external world, visual sampling tends to move to the next visual object before behaviour associated with the previous object is complete^7^, ^8^, ^9^. Our data can be viewed as the *internal* analogue of this ‘looking ahead’ phenomenon, where the internal focus switches to the next-relevant memory content, while behaviour associated with presently relevant content is still ongoing. Indeed, similar to the current data on the prospective, leading nature of our internal focus, switches in perceptual sampling can also lead manual action by half a second^7^ and occur without object visibility^9^.

Our results suggest a form of memory offloading, whereby presently relevant memory content is offloaded to the response-execution system before response completion — allowing the internal focus to switch early and be prepared for what is next. Notably, in our task, there was no need to switch early: report 1 was followed by an imposed one-second delay before probing report 2. Thus, the brain’s modus operandi may be to switch to next-relevant (memory) content whenever it can afford to do so – and our data reveal that this point is reached remarkably early during sequential memory-guided behaviour.

## Supplemental information

Document S1. Experimental Procedures, Author Contributions, Data Accession Link and Two Figures
