# Supplementary material for: Looking ahead in working memory to guide sequential behaviour
Source: Curr Biol. 2021 Jun 21;31(12):R779–80. doi: 10.1016/j.cub.2021.04.063 (PMC8231093; doi:10.1016/j.cub.2021.04.063)
Supplement: Document S1. Experimental Procedures, Author Contributions, Data Accession Link and Two Figures [file mmc1.pdf]

Supplemental Information: Looking ahead in working memory to guide sequential behaviour

Freek van Ede, Jovana Deden, Anna C. Nobre

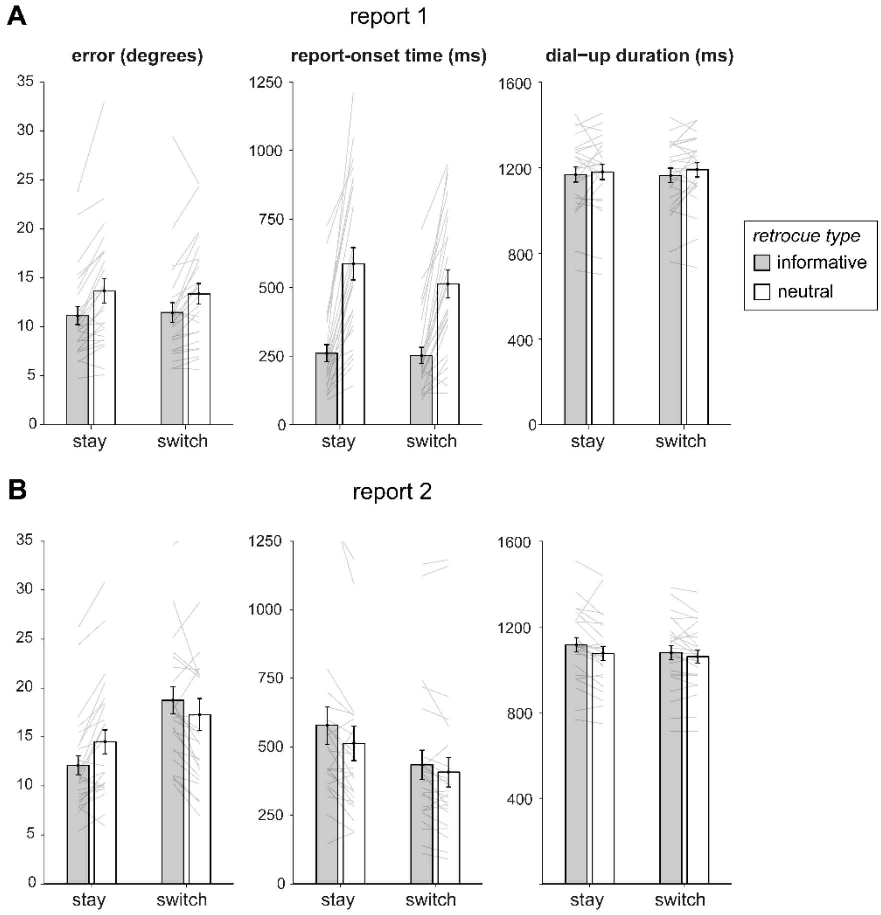

Figure S1. Similar performance benefits from retrocues in stay and switch blocks on report 1. A) Data from the first report after the retrocue (report 1). Error bars represent ± 1 s.e.m. Grey lines show individual participants. In both block types, informative retrocues (compared to neutral retrocues) reduced reproduction errors (stay: $t(24) = -5.014$, $p = 4.013e-5$; $d = -1.003$; switch: $t(24) = -3.69$, $p = 0.001$) and report-onset times (stay: $t(24) = -7.732$, $p = 5.737e-8$; $d = -1.546$; switch: $t(24) = -7.485$, $p = 1.004e-7$; $d = -1.497$), but did not influence the duration of the dial-up reports (stay: $t(24) = -0.678$, $p = 0.504$; $d = -0.136$; switch: $t(24) = -1.709$, $p = 0.1$; $d = -0.34$). Retrocue benefits (informative vs. neutral) did not differ significantly between stay and switch blocks when considering error ($t(24) = -0.883$, $p = 0.386$; $d = -0.177$) but were significantly larger in stay blocks when considering response-onset times ($t(24) = -4.16$, $p = 3.513e-4$; $d = -0.832$). Moreover, when focusing on just the trials with an informative retrocue (those analysed in main Figure 1B,C), we found no significant differences on report 1 between stay and switch trials for any of the dependent variables: error ($t(24) = -0.986$; $p = 0.334$; $d = -0.197$); report-onset times ($t(24) = 0.647$; $p = 0.524$; $d = 0.13$); dial-durations ($t(24) = 0.349$; $p = 0.73$; $d = 0.07$). **B)** For completeness, panel B shows the equivalent data from report 2. This showed, for example, that the cueing benefit on responses errors persisted in stay blocks ($t(24) = -4.894$, $p = 5.452e-5$; $d = -0.978$), while in switch blocks we now found a slight cost of the initial retrocue ($t(24) = -2.514$, $p = 0.019$; $d = 0.503$). This is in line with a modest trade-off for item 2 when having previously prioritised item 1. Also, participants were slightly slower to initiate report 2 in trials in which report 1 had been preceded by an informative vs. neutral retrocue (stay: $t(24) = 3.282$, $p = 0.003$; $d = 0.656$; switch: $t(24) = 2.478$, $p = 0.021$; $d = 0.496$). Data in panels A and B are on the same scales to facilitate visual comparisons.

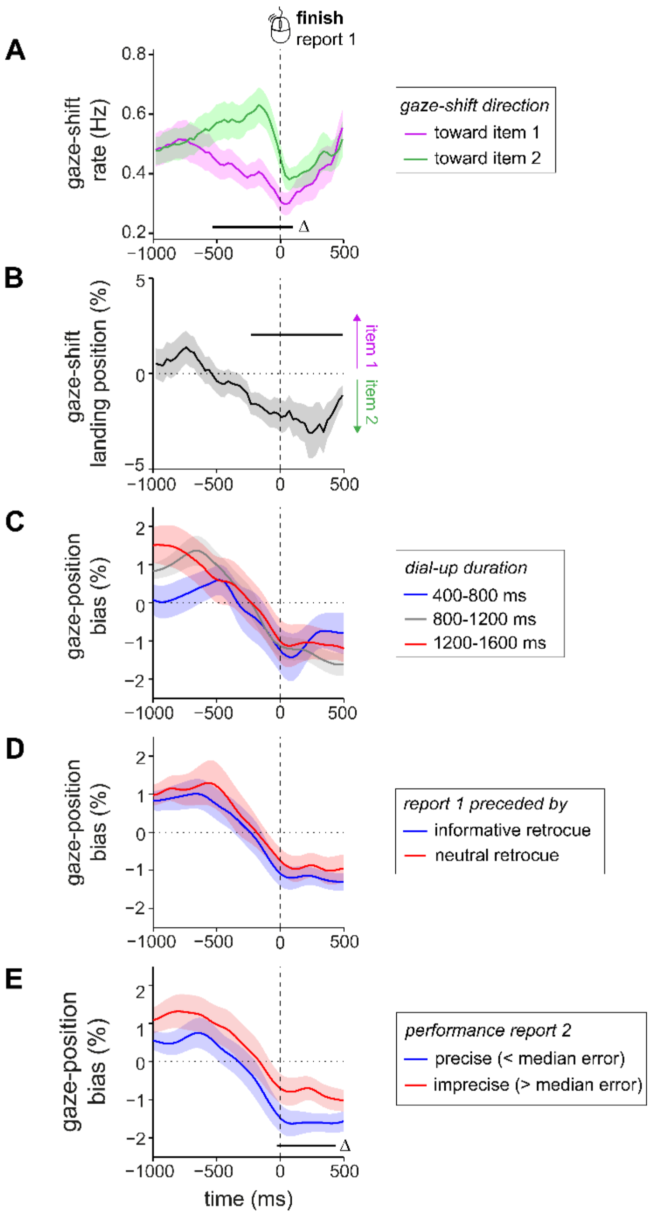

Figure S2. Looking ahead in working memory is also evident in complementary analyses of gaze-shift rates and landing positions (A,B), occurs regardless of dial-up duration and prior anticipation of report 1 (C,D), and predicts performance on report 2 (E). A) Rate of gaze shifts toward the memorised location of item 1 (purple) or item 2 (green), in switch trials aligned to the end of report 1. Gaze shifts were identified using a median threshold on gaze velocity time courses, as described in [S1]. Timecourses were constructed using a 300- ms sliding time window that was advanced over the data in 30-ms steps. In line with the gaze position data presented in main Figure 1B, the rate of gaze shifts – predominantly in the microsaccade range (see inset main Figure 1B) – toward the next-relevant item starts to increase from approximately 500 ms before the end of report 1 completion (cluster $p < 0.0001$). **B)** Average gaze-shift landing position (from all gaze shifts depicted in panel A) also confirms the early nature of this internal priority switch, with average gaze-shift landing positions reversing direction (switching toward the direction of the memorised location of item 2) prior to response 1 completion (cluster $p = 0.002$). **C)** Gaze-position bias in switch trials aligned to the end of the first report for trials with short, medium, and long dial-up durations of report 1 confirm that looking ahead in working memory starts before conclusion of the first report in all cases. **D)** Gaze-position bias in switch trials aligned to the end of the first report, separately for reports preceded by an informative (as in all other plots) or a neutral retrocue. In trials with a neutral retrocue, participants do not yet know the order in which the items will be probed (in switch blocks) until the first reporting display appears. **E)** Gaze-position bias in switch trials aligned to the end of the first report, separately for trials with a precise vs. imprecise memory reproduction at report 2. Precise and imprecise trials were separated based on a median split of response errors at report 2, after regressing out reproduction errors to report 1 (to remove global difference in performance due to factors like arousal or motivation). Specifically, we used a general linear model to predict reproduction errors at report 2 from the errors at report 1, and subsequently performed a median split on the residuals. Gaze bias around the time of the priority switch is significantly more directed at item 2 in trials with a more precise report of item 2 (cluster $p = 0.021$). Shading represents ± SEM. Horizontal lines denote significant clusters. Time zero denotes completion of report 1.

Supplemental Experimental Procedures

Participants

The study was approved by the Central University Research Ethics Committee of the University of Oxford. Twenty-five healthy human volunteers (14 female, 3 left-handed, age-range 19-37, M = 26) participated in the study. Participants provided informed consent prior to participation and were compensated £10/hour.

Task and procedure

Participants performed a visual working memory task with two sequential reports (Figure 1A). Participants sat approximately 95 cm from the monitor (22-inch; 1680 × 1050 pixels; refresh rate 100 Hz). Participants were instructed at the beginning of the experiment to maintain fixation on the central fixation cross (but fixation was not controlled by means of online feedback during the experiment).

Encoding displays consisted of one green (RGB: 133, 194, 18; measured CIE xyY: 0.37, 0.56, 38.3) and one purple (RGB: 197, 21, 234; measured CIE xyY: 0.27, 0.11, 13.64) oriented bar. Bar orientations were drawn independently for both memory items (with no criterion for a minimal difference between them) and could each span the full range (0-180 degrees). Bars were anchored at 5.7 degrees visual angle (dva) to the left and right of fixation and were each approximately 5.7 dva in length, and 0.8 dva in width (as in [S1,S2]). The central fixation cross spanned an area of approximately 0.4 dva in width and height. Colour-location mappings of the bars varied across trials.

Bars were presented for 250 ms followed by a 750 ms delay period (in which only the fixation cross remained in the screen) before the presentation of a retrocue (250 ms duration) that was either informative (80%) or neutral (20%). Informative retrocues involved a colour change of the central fixation cross that matched the colour of either the green or purple bar (in memory) and predicted with 100% reliability that this bar would be probed for report first. Neutral retrocues involved a colour change to grey (RGB: 100, 100, 100; measured CIE xyY: 0.3, 0.31, 11.77). Trials with informative and neutrals retrocues occurred intermixed. Informative retrocues equally often cued the left/right bar and the green/purple bar.

Memory probes consisted of a fixation-cross colour change that remained present until response completion. Participants were asked to report back the precise orientation of the colour-matching memory representation (as in [S1,S2]). The central reporting dial (5.7 dva in diameter) appeared upon initial displacement of the computer mouse, after which the dial-up report remained controlled by the mouse. Dial-up was terminated by a click of the left mouse button. Participants had unlimited time to initiate their report. Once initiated, participants had a maximum of 2500 ms to complete their report. We only considered trials in which response-onset time for the first report was within 4 SD of the mean (98 ± 0.25 % of trials) and in which participants terminated their first report before the maximum response time (98.4 ± 0.27 % of trials).

Each trial required two reports. Report 1 was probed 2000 ms after retrocue onset. Report 2 was probed 1000 ms after completion of the first report. Sequential memory demands were varied across blocks. In “stay blocks” the same bar was probed twice. In “switch blocks” one bar was probed for report 1 and the other bar for report 2. In trials with an informative retrocue, the cued memory item was always probed first. The second report was followed by feedback of the average precision of both reports, in the form of a number between 1 (maximum error) and 100 (perfect report), presented above the fixation cross (200 ms duration). After feedback offset, the next trial would start 500 to 800 ms later.

Stay and switch blocks were interleaved with never more than two blocks of the same type in a row. Block type was indicated prior to each block and remained printed on the top of the screen throughout the block (with “cued twice” denoting stay blocks and “cued-uncued” denoting switch blocks). In total, participants completed 12 stay and 12 switch blocks of 30 trials each (720 trials in total). The study lasted approximately 90 minutes.

Eye-tracking acquisition and analysis

Gaze was tracked using an EyeLink 1000 (SR Research) mounted on the table in front of the participant. Gaze position was tracked for both eyes simultaneously, at a sampling rate of 1000 Hz. Prior to the study, the eye tracker was calibrated using EyeLink’s default calibration software. In addition, after every two experimental blocks, a custom calibration module was inserted in which participants were asked to sequentially look at seven positions (left-top, right-top, left-middle, centre, right-middle, left-bottom, right-bottom) that matched the eccentricities of the bars in the study (5.7 dva from the centre). Gaze-position data from custom calibrations were used to normalise horizontal gaze position during the task; i.e. to express gaze-position biases in percentage, with 0 denoting central fixation and ±100 % denoting the centre of each bar.

Eye-tracking data were analysed in Fieldtrip [S4]. Data were separately segmented relative to the retrocue onset and to the completion of report 1. We focused on gaze-position biases along the horizontal axis and (for all but one analysis) only considered trials with an informative retrocue (80% of all trials). Trials with a neutral retrocue served primarily to evaluate the behavioural benefits of retrocues in stay and switch blocks.

Gaze towardness – our primary index of the gaze bias of interest [S1,S2] – was constructed by comparing calibration-normalised horizontal gaze position in trials in which the colour retrocue matched the left vs. the right bar in memory (i.e., [right-left] / 2). Left and right were defined only relative to the original bar locations during encoding (retrocues and reporting dials were always presented centrally). Positive towardness values signify a directional bias toward the memorised location of the cued representation, whereas negative towardness values signify a directional bias toward the other representation. No baseline corrections were applied to the gaze bias time course data. After the calibration-normalisation (that was applied to all trials and time points equally) gaze-position data were defined relative to the fixation cross at the centre of the screen.

Because we previously established [S1] that the directional gaze bias of interest consists of a directional bias of fixational eye-movements (microsaccades [S3,S5-S7]), we only included trials in our gaze-position analyses in which gaze position during the trial did not deviate more than ±50 % from the centre (which was the case in the vast majority of trials: 96.5 ± 0.4 % of trials; see also inset Figure 1B). Note that this also distinguishes the current gaze bias from previous reports of ‘looking-at-nothing’ [S8-S10], as discussed more extensively in our prior report that was dedicated to the nature of this bias [S1]. Gaze-position towardness time courses were smoothed with a Gaussian kernel with a s.d. of 50 ms.

As in our prior reports [S1,S2], we statistically evaluated gaze towardness time courses using cluster-based permutation analyses [S11] that circumvent the multiple comparisons problem by evaluating the full time axis under a single permutation distribution of the largest cluster. We used default cluster settings in Fieldtrip. Timing of gaze biases after the retrocue and during the priority switch were determined on the temporal derivative of the towardness time courses (i.e. finding when the respective gaze-position biases showed the largest rate of change).

For visualisation purposes, we also calculated two-dimensional heatmaps of gaze density (as in [S1,S2]). We did this separately for different time windows, aligned to completion of report 1. We compared heatmaps in switch trials as a function of bar location, such that positions for which gaze density was

higher toward the first-relevant memory representation were coded in purple (and toward the left) while positions for which gaze density was higher for the second-relevant memory representation were coded in green (and towards the right).

In addition to our main analyses that focused on gaze position, we also performed complementary analyses of gaze shifts. Gaze shifts were identified using a median threshold on gaze velocity time courses, as also described in [S1]. We considered gaze shifts for which the rate of change in gaze position exceeded 10 times the median velocity, and calculated gaze-shift magnitudes as the difference in horizontal gaze position in the period before (-100 to -50 ms) vs. after (50 to 100 ms) this threshold crossing.

Author Contributions

F.v.E. and A.C.N. designed the study, F.v.E. and J.D. conducted the experiment, F.v.E. performed the analyses and drafted the figures, F.v.E. and A.C.N. wrote and revised the manuscript.

Supplemental Data Accession Link

Anonymised behavioural and eye-tracking data associated with this manuscript have been made publically available through Zenodo and can be accessed at:
https://doi.org/10.5281/zenodo.4700923

Supplemental References

S1. van Ede, F., Chekroud, S.R., and Nobre, A.C. (2019). Human gaze tracks attentional focusing in memorized visual space. Nat. Hum. Behav. 3, 462–470.

S2. van Ede, F., Board, A.G., and Nobre, A.C. (2020). Goal-directed and stimulus-driven selection of internal representations. Proc. Natl. Acad. Sci. 117, 24590–24598.

S3. Rolfs, M. (2009). Microsaccades: Small steps on a long way. Vision Res. 49, 2415–2441.

S4. Oostenveld, R., Fries, P., Maris, E., and Schoffelen, J.M. (2011). FieldTrip: Open source software for advanced analysis of MEG, EEG, and invasive electrophysiological data. Comput. Intell. Neurosci. *2011*, 156869.

S5. Engbert, R., and Kliegl, R. (2003). Microsaccades uncover the orientation of covert attention. Vision Res. *43*, 1035–1045.

S6. Hafed, Z.M., and Clark, J.J. (2002). Microsaccades as an overt measure of covert attention shifts. Vision Res. *42*, 2533–2545.

S7. Corneil, B.D., and Munoz, D.P. (2014). Overt responses during covert orienting. Neuron *82*, 1230–1243.

S8. Richardson, D.C., and Spivey, M.J. (2000). Representation, space and Hollywood Squares: Looking at things that aren't there anymore. Cognition *76*, 269–295.

S9. Ferreira, F., Apel, J., and Henderson, J.M. (2008). Taking a new look at looking at nothing. Trends Cogn. Sci. *12*, 405–410.

S10. Johansson, R., and Johansson, M. (2014). Look here, eye movements play a functional role in memory retrieval. Psychol. Sci. *25*, 236–242.

S11. Maris, E., and Oostenveld, R. (2007). Nonparametric statistical testing of EEG- and MEG-data. J. Neurosci. Methods *164*, 177–90.
